# Identification of a new strain of mouse kidney parvovirus associated with inclusion body nephropathy in immunocompromised laboratory mice

**DOI:** 10.1080/22221751.2020.1798288

**Published:** 2020-08-10

**Authors:** Zhongming Ge, Sebastian E. Carrasco, Yan Feng, Vasudevan Bakthavatchalu, Damodaran Annamalai, Robin Kramer, Sureshkumar Muthupalani, James G. Fox

**Affiliations:** Division of Comparative Medicine, Massachusetts Institute of Technology, Cambridge, MA, USA

**Keywords:** MKPV, genome organization, IBN, PCR diagnosis

## Abstract

Inclusion body nephropathy (IBN) and kidney fibrosis in aged immunodeficient mice and, to lesser extent, in immunocompetent mice have been recently linked to infection of mouse kidney parvovirus (MKPV), also known as murine chapparvovirus (MuCPV). Knowledge about its prevalence and the complete genome sequence of more MKPV strains is essential for understanding phylogenetic relationships and pathogenicity among MKPV strains. In the present study using PCR and genome walking, we determined the complete 4440-nucleotide genome of a new MKPV strain, namely MIT-WI1, which was identified in IBN-affected *Il2rg^−/–^Rag2^−/–^ c-Kit ^W–sh/W–sh^* mice housed in the vivarium at Whitehead Institute for Biomedical Research (WI). The overall nucleotide (>94%) and deduced amino acid sequences (>98%) of p10, p15, NS1 (replicase), NS2 and VP1 (capsid protein) within the MIT-WI1 genome, are closely related to MKPV/MuCPV strains described in laboratory and wild *Mus musculus* mice. In addition, PCR and qPCR assays using newly designed primers conserved among the known MKPV/MuCPV genomes were developed and utilized to assess MKPV status in selected laboratory mice. MKPV was also detected in immunodeficient (NSG) and immunocompetent (Crl:CD1(ICR), UTX^flox^) mouse strains/stocks. The abundance of the MKPV genome copies was significantly correlated with the severity of IBN. Our data indicate that MKPV is present in selected mouse strains/stocks, and provides new insights into the genome evolution of MKPV.

## Introduction

It has been documented that intranuclear inclusion body nephropathy (IBN) occurs in aging laboratory mice [1]. IBN has been reported predominantly in immunodeficient mice but also in aged immunocompetent mice, including inbred C56BL/6 and outbred Crl:CD1(ICR) mice; however, its aetiology was undetermined until recently [2–5]. In 2018, Roediger et al. reported that IBN in mice was induced by infection with an atypical parvovirus, termed mouse kidney parvovirus (MKPV). This newly recognized parvovirus is highly divergent from previously known mouse parvoviruses (e.g. mouse pavrovirus-1 and minute virus of mice) that also infect laboratory mice [5]. MKPV has been currently placed in the provisional virus genus *Chapparvovius*. Despite that no virions have been identified, the MKPV genome and clinical phenotype was transmitted to healthy mice by co-housing with the MKPV-infected mice. Abundance of MKPV genomic DNA in kidney, serum and urine of the co-housed mice increased in aging mice [5]. In addition, the genomes of MKPV strains isolated from laboratory mice housed at the Centenary Institute Animal Facility, Australia, and at the Memorial Sloan Kettering Cancer Center, New York share over 98% nucleotide sequence similarity [5]. Furthermore, a viral genome displaying over 97% sequence similarity to the MKPV (also known as [aka]murine chapparvovirus) genome were detected in 38.2% of wild *Mus musculus* mice collected from the urban areas of New York City, where there was a relatively higher prevalence (65%) for adult mice compared with sub-adult mice (11%) [6].

C57BL/6J *c-Kit^W–sh^* mice, deficient in mast cells, have been utilized at Whitehead Institute for Biomedical Research (WI), Cambridge, Massachusetts to examine how human neural crest cells contribute to embryonic and post-natal mouse development [7]. To improve human cell incorporation into the *c-Kit^W–sh^* mice and also to establish a system for human disease modelling, CRISPR (clustered regularly interspaced short palindromic repeat) and Cas (CRISPR-associated) proteins were applied to knockout interleukin 2 receptor subunit gamma (*Il2rg*) and recombination activating gene-2 (*Rag2*) in the *c-Kit^W–sh^* mice, to create *Il2rg^−/–^Rag2^−/–^c-Kit ^W–sh/W–sh^* mice (aka *Il2rg^−/–^Rag2^−/–^W^sh^/W^sh^*). This newly created *Il2rg^−/–^Rag2^−/–^c-Kit ^W–sh/W–sh^* mouse strain developed unexpected morbidity and mortality associated with infection of *pks^+^ Escherichia coli*, urosepsis and meningitis [8]. In the present study, we identified and determined the complete genome sequence of a new strain of MKPV infecting *Il2rg^−/–^Rag2^−/–^c-Kit ^W–sh/W–sh^* mice and selected additional mouse strains/stocks. Moreover, we evaluated histological lesions consistent with IBN in the kidneys of these mice. Our findings have provided evolutionary insights into the transmission of MKPV. Further, PCR assays have been established for detecting MKPV in clinical and environmental samples.

## Materials and methods

*Mouse strains and sampling.* C57BL/6 *c-Kit^W–sh^* (B6.Cg-*Kit^W–sh^*/HNihrJaeBsmJ, Stock No:030764) mice were obtained from the Jackson Laboratory and maintained in the WI animal facility which is affiliated with Massachusetts Institute of Technology (Cambridge, MA, USA). C57BL/6 *Il2rg^−/–^Rag2^−/–^cKit^W–sh^* mice, which is represented by B6.Il2rRag2W throughout the text, were generated via one-cell embryo injection of Cas9 mRNA and sgRNA into C57BL/6 *c-Kit^W–sh^* mice as previously described [8]. Additional laboratory mouse strains/stocks used in the present study included immunodeficient NSG (NOD.Cg-*Prkdc^scid^ Il2rg^tm1Wjl^*/SzJ, Stock No: 001976) and immunocompetent UTX^flox^ (B6;129S-*Kdm6a^tm1.1Kaig^*/J, stock No:024177) from Jackson Laboratory and Crl:CD1(ICR) mice from Charles River Laboratories. All mice were free of murine pathogens such as *Helicobacter* spp., *Citrobacter rodentium*, *Salmonella* spp., endoparasites, ectoparasites, and exogenous murine pathogens including mouse parvovirus 1 (MPV1) and minute virus of mice (MVM). The mice were housed under speciﬁc pathogen-free conditions in AAALAC International-accredited facilities in static microisolater cages, fed irradiated diet and provided water ad libitum. All animal housing conditions and experimental protocols were approved by the MIT Committee on Animal Care. At necropsy, kidney tissues collected from these mice were embedded in paraffin or snap-frozen in liquid nitrogen followed by storage at −80°C.

*DNA extraction from formalin-fixed paraffin-embedded (FFPE) tissues, fresh murine tissues and faecal samples.* Longitudinal sections of the left kidney and/or transverse sections of the right kidney were embedded in individual cassettes for each case and were sectioned at 15-µm thickness. Instruments and the faces of the microtome plate and FFPE blocks were cleaned with 0.1% hydrochloric acid followed by a treatment with 100% ethanol between samples. Microtome blades were changed every other block. Blank blocks (controls) were used between samples to assess the possibility of cross-contamination. All control blocks were PCR-negative for MKPV. The sectioned tissues were deparaffinized by incubation in 1-mL xylene for 10 min on a shaker with agitation at 60 rpm, followed by centrifugation at 14,000×g for 5 min, and then xylene was carefully removed. Kidney tissues were subsequently incubated in 1 mL ethanol for 5 min at room temperature, and centrifuged at 14,000×g for 5 min followed by careful removal of ethanol and air-dry. Total DNA from deparaffinized kidneys was prepared using the QIAamp DNA FFPE Tissue Kit following the manufacturer's instruction with some modifications. Briefly, after incubation with tissue lysis buffer ATL and proteinase K at 56°C, additional 180 µl Buffer ATL was added to the samples which were then incubated at 70°C for 10 min. DNA was eluted with 50 µl ddH_2_O, and the concentration and quality of the prepared DNA samples were evaluated using the Nanodrop ND-1000C (Thermo Scientific, Rockford, IL). The resulting DNA was reconstituted in 1×TE buffer and stored at −20°C prior to use. Total DNA from frozen kidneys and murine faeces were extracted using QIAamp DNA Mini Kit and QIAamp Stool DNA Mini Kit following the manufacturer's protocols respectively.

*PCR-based MKPV genome walking.* Nucleotide sequences of MKPV PCR primers and a qPCR probe are listed in Table 1. PCR was generally performed in a 25-µl reaction volume using the Expand High Fidelity PCR System according to the manufacturer's instruction (Sigma-Aldrich Corp. St. Louis, MO). A PCR program consists of 5 min at 95°C, thermocycling (1 min at 95°C, 1 min at 55°C, 1 min at 72°C)×35, and 4 min at 70°C, followed by incubation at 4°C. The double-strand nucleotide sequences of all the PCR products covering the genomes of MKPV isolates were determined using Sanger sequencing by Quintara Biosciences (Cambridge, MA).

**Table 1.** Nucleotide sequences of PCR primers and a Taqman probe for detecting MKPV.

Primer ID	Nucleotide sequence (5′→3′)	Strand
	Genome walking	
F12	AGCAGTCATGTGTTGTTGTCCTTTGT	Sense
R13	ACGTTCCATTTGCGCTTGCATT	Antisense
F2b	ACCGGACACTTCCTTTGACAGCG	Sense
R2	AGCTGGTGAAATTCCTTGGTGTA	Antisense
F3	CATCCACCCGAGCCAGAAGCCAT	Sense
R3	TAGTTGAGGAGCCGGTGCTGCA	Antisense
F4	GCACCCACAGCCCAAGACCTAG	Sense
R4	CATGAGTATATGGCCATATG	Antisense
F5	ACAAAAATCTCAGTGGTTCATG	Sense
R5	TTGGTGCATAGATGCGGCTGCGT	Antisense
F10	TATGGACCTACACAATGCT	Sense
R15	GTTAACAATAAGATTTTGTATTGTG	Antisense
	5′- and 3′-ITR regions	
F1	CTACGAAACTGGGCAGGGCGC	Sense
R16	CCTTTGCATGTGCGCGCACT	Antisense
F14	TTGTTAACATGTGTTAACGTTAAC	Sense
R12	GCATGGCGTACCACTGCCTGTA	Antisense
F15	GCATGTACCTCAACCATAAG	Sense
R14	CCACATGTTCTGTACGACT	Antisense
F16	CCCAGTCGTACAGAACA	Sense
R6	TTAACCCGCCTCCCAATCTTGGCT	Antisense
	PCR and qPCR	
MKPV PCR-rF	TGTCAGATATAGAAGAACCAGATC	Sense
MKPV PCR-rR*	GGTGCTGCTTTCATGCAGTCTTCTA	Antisense
MKPV-qF	CCACCTCACAGATGCATACTATCA	Sense
MKPV-qProbe	FAM-AGCTGARATCACAGAAGTSATTATGCAACA-MGB	Sense
MKPV qR	The same as *	Antisense

*DNA cloning and development of qPCR.* To construct a recombinant plasmid for use as MKPV qPCR standard, a PCR fragment corresponding to nucleotides 769–1656 of the MIT-WI genome was amplified with a primer pair F2b/R2 and then cloned into the TOPO vector pCR^TM^ 2.1 according to the supplier's protocols (ThermoFisher Scientific). DNA sequence authenticity of the insert was confirmed by Sanger sequencing (Quintara Biosciences). A qPCR standard curve of 10-fold dilutions from 10^7^ to 10 copies of the recombinant plasmid DNA was generated. qPCR assays were performed in a 10-µl volume consisting of 5 µl 2 X Fast Universal Mixture (ThermoFisher Scientific), 3 µl DNA template, 1 µl of the mixture containing forward/reverse primers and the probe (2 µM each) in the 7500 Fast Real-Time PCR system (Thermo Fisher Scientific) with the default setting. The genome copies of MKPV in the samples were normalized to murine DNA which was quantified using the 18S rRNA gene-based primers/probe (ThermoFisher Scientific) and expressed per pg and µg of murine DNA for FFPE and frozen kidney tissues, respectively. A lower quantity of mouse DNA was used for the FFPE tissues due to the decreased yield of murine DNA.

*Histological evaluation*. Detailed necropsies were performed on immunocompromised and immunocompetent mice that were either found dead or following CO_2_ euthanasia*.* Tissues were placed in 10% neutral-buffered formalin for at least 48 h. After routine paraffin embedding of the fixed tissue, sections were cut at 5 μm thickness and stained with haematoxylin–eosin. A subset of histologic sections was stained with Masson's trichrome to confirm the presence of collagen deposition and fibrosis in the kidneys of these mice. To assess the degree of IBN in the kidneys of B6.Il2rRag2W and NSG mice, hemisected kidneys were examined at 200–400X magnification for foci tubular loss, atrophy, degeneration, regeneration and necrosis, karyomegaly and intranuclear inclusions in tubular epithelial cells, interstitial fibrosis, papillary necrosis, and leukocyte infiltration. The presence of intranuclear inclusion bodies in tubular epithelial cells from kidneys with and without lesions was evaluated in five random fields at 400X magnification. One or two hemisected kidneys were graded using a scale of 0–4, in which a score of 0 represented no IBN; 1 = < 10% of the renal sections had IBN lesions; 2 = 10–25% of the renal sections had IBN; 3 = 25–75% had IBN; 4 = extensive IBN lesions involving > 75%.

Non-IBN lesions for kidneys of aged UTX^flox^ mice were scored by severity from 0 to 4, in which a score of 0 represented normal; 1 = <10% renal parenchyma affected by lesions; 2 = 10–30% renal parenchyma affected by lesions; 3 = 30–70% affected by lesion; 4 = > 70% renal parenchyma affected by lesions [9]. The following lesions were scored using this criterion: nephropathy, pyelonephritis/pyelitis, and lymphoid aggregates. The category of nephropathy included chronic glomerular changes with or without tubular proteinosis, tubular degeneration/regeneration, interstitial inflammation, and fibrosis [9]. Other renal lesions were scored as present (1) or absent (0) included mineralization, hydronephrosis and tumour.

*In situ chromogenic detection of MKPV RNA.* We performed RNAscope on IBN-affected and MKPV-free renal tissues of B6.Il2rRag2W and NSG mice since this assay was previously used to detect MKPV mRNA *in situ* in the kidney tissues of NSG mice with IBN[5]. In the present study, hybridization probes were designed to target a 978-base sequence of MKPV MIT-WI1 mRNA covering both VP1 and NS1 regions (nucleotides 2442–3419 of the viral genome) (Advanced Cell Diagnostics [ACD], Newark, CA). The assay was performed in ACD HybEZ II Hybridization System using the RNAscope 2.5 HD Reagent Kit-Red following the ACD protocols. Positive (mouse PPIB) and negative (dapB) control probes in lieu of the target probe were used on the same specimen for validating the assay conditions (ACD). The stained slides were examined by a board-certified veterinary pathologist (S.C).

*Statistical analyses.* DNA sequences and deduced amino acid sequences were analyzed using the DNAStar Lasergene package. Linear regression and Pearson correlation between abundance of MKPV and severity of IBN were performed using the GraphPad Prism 5 Package. *P* values of < 0.05 were considered significant.

## Results

### MKPV is associated with IBN in a colony of B6.Il2rRag2W mice

We recently reported the occurrence of a sudden increase in morbidity and mortality in a colony of B6.Il2rRag2W mice associated with *pks^+^ E. coli*-induced urosepsis and meningitis [8]. Retrospective evaluation of the kidneys from 14 of 15 B6.Il2rRag2W mice (5.5–22 months old) in this study [8] confirmed the presence of interstitial fibrosis and intranuclear inclusion bodies in convoluted tubules in 10 of 14 mice; these lesions are consistent with IBN described recently [5]. An additional 10 B6.Il2rRag2W mice (3.5–10 months old) submitted for necropsy after the *pks+ E. coli* outbreak also had IBN. Of these 24 mice, 10 had additional lesions consistent with suppurative pyelonephritis, pyelitis or nephritis. Hydronephrosis was present in 60% (12/20) of the mice with IBN lesions, and 5 of these 12 cases also had suppurative pyelonephritis.

**Figure 1.**
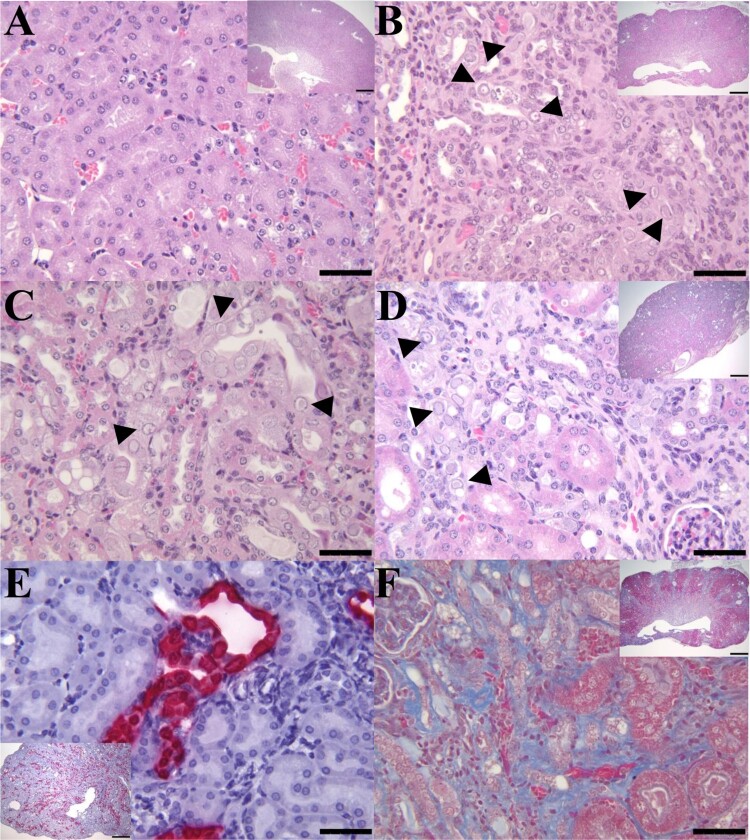
IBN in the kidneys of B6.Il2rRag2W mice. (A) Representative H&E section of a kidney (400×; inset 20×) from an MKPV-negative mouse. (B) Longitudinal section of kidney from case 1 with chronic IBN and mild hydronephrosis (inset 20×) and mild multifocal neutrophilic pyelonephritis. The kidney has an undulating irregular renal cortex, containing multifocal to coalescing areas of interstitial fibrosis, tubular loss, and tubular degeneration with single cell necrosis, karyomegaly, and intranuclear amphophilic to eosinophilic inclusions (arrowheads: 400×). (C) Longitudinal section of kidney from case 2 with IBN and mild neutrophilic pyelonephritis. Affected convoluted tubules in the renal cortex had vacuolated and degenerated epithelial cells with karyomegalic nuclei and large (∼5–6 µm) amphophilic intranuclear inclusions or small (∼1 µm) densely eosinophilic intranuclear inclusions (arrowheads: 400×). (D) Longitudinal section of kidney from case 3 with moderate to severe IBN (inset 20×). High magnification of the kidney from case 3 exhibited areas of interstitial fibrosis and tubular degeneration with karyomegalic nuclei, chromatin margination and intranuclear inclusions (arrowheads: 400×). (E) MKPV in situ hybridization signals (RNAscope) in renal tubules of case 3 (inset 40×). MKPV nucleic acids were detected (fast red staining) in the nucleus and cytoplasm of affected tubular epithelial cells (400×). (F) Masson trichrome staining of an affected kidney (case 1) showed marked collagen deposition (blue staining) in the renal interstitium (400×; inset 20×).

Compared with a healthy kidney (Figure 1(A)), IBN lesions from a B6.Il2rRag2W mouse (case 1) with concurrent mild neutrophilic pyelonephritis and hydronephrosis were characterized by multifocal areas of glomerular and tubular loss, tubular atrophy, necrosis and degeneration with karyomegaly and intranuclear inclusions, and interstitial fibrosis (Figure 1(B,F)) in the renal cortices and inner and outer stripe medullary regions. The renal papilla and inner medullas of case 1 also exhibited multifocal areas of interstitial fibrosis, tubular degeneration and necrosis mixed with mild neutrophilic pyelonephritis. Affected cortical tubules of case 1 and an additional B6.Il2rRag2W mouse (case 2) with mild neutrophilic pyelonephritis frequently had vacuolated tubular epithelial cells with pale-eosinophilic, granular cytoplasm and karyomegalic nuclei with peripheralized chromatin and large granular amphophilic intranuclear inclusions (Figure 1(B,C)). Some tubular epithelial cells had karyomegalic nuclei with peripheralized chromatin and small dense eosinophilic intranuclear inclusions (Figure 1(B,C)). In addition, 13 B6.Il2rRag2W mice had lesions consistent with IBN, which was attributed as the cause of death in these mice. Similarly to the previous cohort of cases, IBN (case 3) was characterized by multifocal areas of interstitial fibrosis (Figure 1(D)), tubular loss, necrosis and/or degeneration with karyomegalic nuclei, chromatin margination and the presence of intranuclear inclusion bodies (Figure 1(D)).

Given that there were histological similarities of IBN in B6.Il2rRag2W with those noted in other immunocompromised mice (e.g, NSG, *Rag1^−/–^*) [5], we performed PCR to examine the status of MKPV infection in the FFPE kidneys of B6.Il2rRag2W mice using a primer set targeting the *NS1* of MKPV described previously [5]. A 311-bp PCR product was amplified from 20 mice with IBN and 3 mice without IBN, but with lesions secondary to sepsis. One mouse with suppurative pyelonephritis was negative for MKPV. The nucleotide sequences of PCR amplicons displayed 98% and 99% similarity to the corresponding regions of MKPV detected in mice at the Centenary Institute and MSKCC, respectively.

Next, we performed chromogenic in situ hybridization on sections of FFPE kidneys from mice with and without IBN using RNAscope with the probes complementary to the MKPV MIT-WI1 mRNA partially covering both *VP1* and *NS1* regions (nucleotides 2442–3419 of the viral genome, see below). MKPV mRNA was distributed throughout the nucleus and cytoplasm of tubular epithelial cells in the kidneys of IBN-affected B6.Il2rRag2W mice (Figure 1(E)) (case 3) and (sFigure 1B) (an additional mouse with less severe IBN). In contrast, the MKPV mRNA was not detected in a MKPV-negative mouse without IBN (sFigure 1A). These results demonstrated the existence of MKPV in mice maintained in our facility and provided molecular and histopathological evidence that MKPV was likely the causative agent of IBN diagnosed in this colony of B6.Il2rRag2W mice.

### Characterization of the complete genome of a new MKPV strain in B6.Il2rRag2W mice

**Figure 2.**
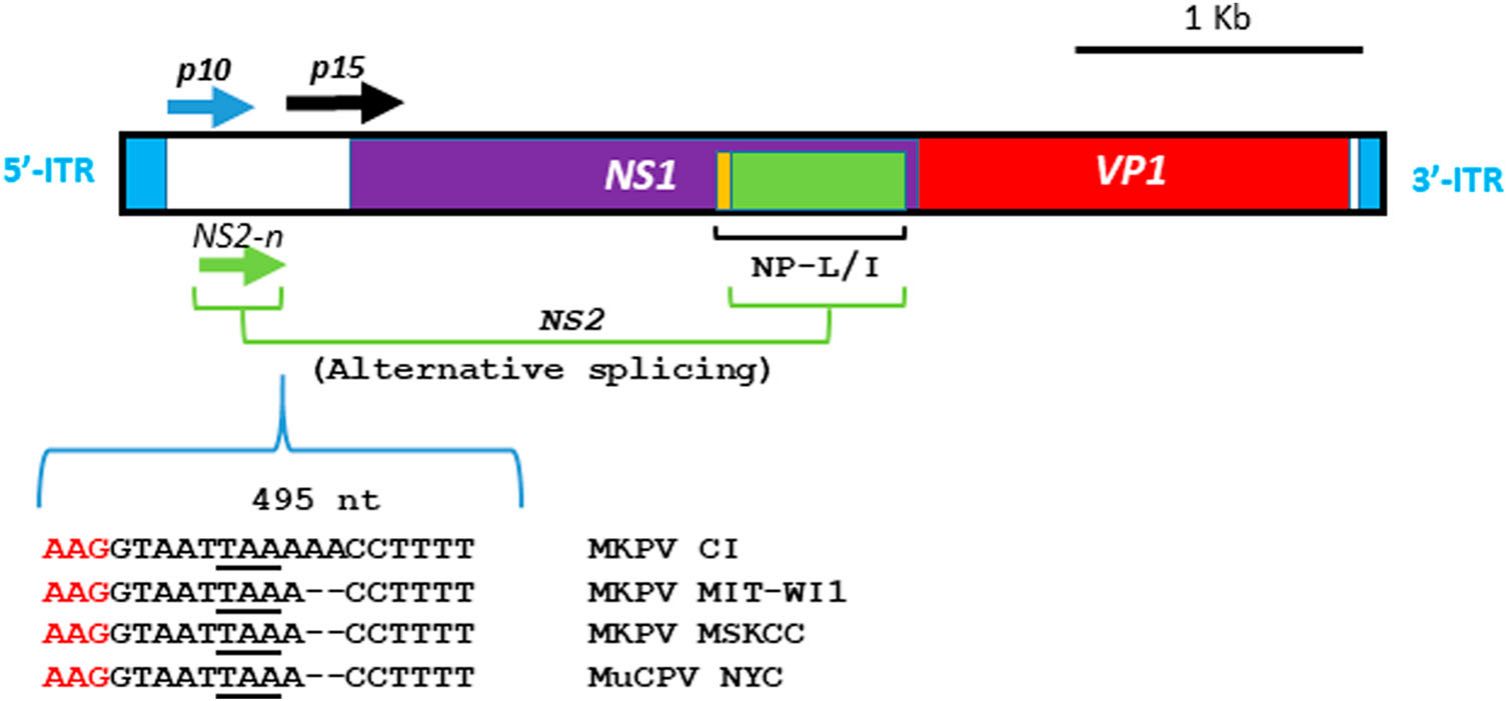
Gene organization of MKPV MIT-WI1. The 4440 nt genome of MIT-WI1 with 5′ and 3′ ITRs contains ORFs which are predicted to encode NS1 (viral replicase), VP1 (capsid protein), accessory proteins p10, p15 and NP-L/I embodied in NS1. NS-2 is formed via alternative slicing which joins the N-terminal ORF (NS-2n in green) and the partial aa sequence of NP-L/I (in green) [5]. This gene organization is conserved among known MKPV/MuCPV strains [10]. There is a dinucleotide deletion at nucleotide 495 in the MIT-WI1 as well as MKPV MSKCC and MuCPV NYC compared with MKPV CI [10].

One complete and two nearly complete genome sequences of MKPV strains were initially deposited in the NCBI database in October 2018; the new versions of these genome sequences have been updated recently (Table 2). For a simplified description, the abbreviations of the respective chapparvoviruses are presented in Table 2; these are used throughout the text. Based on the conserved regions of these MKPV genomes, sequential PCR primers were designed to determine the complete genome sequence of MKPV MIT-WI1 strain via PCR-based genome walking (Table 1). The approximately 4.1 kb sequence of this strain was determined by sequencing of the amplified PCR products. However, it was difficult to amplify single PCR products covering the intact inverted tandem repeats (ITRs) in the 5′ and 3′ terminal regions of the MKPV genome presumably due to their high GC content and stringent stem-loop structures. New primers complementary to the predicted loops were designed and were used to produce overlapping amplicons covering the intact ITRs (Table 1, Figure 2). The complete genome sequence of MKPV MIT-WI1 strain is 4440 nucleotides (nt) in length and has been deposited in the GenBank database with the accession # of MT093738 at the National Center for Biotechnology Information.

**Table 2.** Nucleotide sequence similarities between MIT-WI1 and known MKPV/MuCPV genomes.

Strain	Origin	Host	% nt Sequence similarity^a^	Accession #
MKPV MIT-WI1	MIT and WI MA	Mice	100 (4440 nt. complete)^b^	MT093738^d^
MKPV MSKCC	Memorial Sloan Kettering Cancer Center NY	Mice	99.9 (4315 nt, partial)	NC_04083.1^e^
MKPV CI	Centenary Institute Sydney Australia	Mice	98.2 (4442 nt, complete)	MH670588.2^e^
MuCPV NYC	New York City	Wild *Mus musculus*	98.2 (4174 nt)	MF175078.1^f^
MuCPV XJC	Xingjiang China	Rodents^c^	94.5 (1165 nt, partial)	MG679365.1^g^
TdCPV2	Tasmania Australia	Tasmanian devil	74.4 (4262 nt, partial)	MK513529.1^g^

Notes: ^a^compared with the MIT-WI1 genome.

^b^Number of nucleotides (nt) in each of either complete or partial genomes.

^c^Lee et al. decribed as wlid *Mus musculus* mice (10).

^d^This study.

^e^Ref. [5].

^f^Ref. [6].

^g^Unpublished.

To characterize clonality of this virus in B6.Il2rRag2W mice, the genome sequences excluding their ITR regions of MKPVs from additional 3 FFPE kidneys collected from individual B6.Il2rRag2W mice over a period of 5 years were PCR-amplified and sequenced by Sanger sequencing, which displayed 100% nt sequence identity to MKPV MIT-WI1. The gene organization of the MKPV MIT-WI1 is predicted to be identical to that in the MKPV CI genome, including 5′- and 3′-terminal stem-loop structures and open reading frames (ORFs) for p10, p15, NS1, NS2 (previously NS2-P), NP-L/I (previously NS-L) and VP1, respectively (Figure 2) [5,10].

### Evolutionary relationship

Phylogenetic relationships among members of *Parvoviridae*, mostly based on the amino acid (aa) sequences of NS1 or VP1, have been recently examined [10,11]. We focused on genome comparison of MKPV/MuCPV strains, and the TdCPV2 (Tasmania devil-associated parvovirus 2) genome was used as a distant outliner. The MIT-WI1 genome was subjected to a search in the NCBI database via Blastn. The 3 nearly complete genomes of MKPV/MuCPV and an 1.1 kb *rep* (NS1) sequence of chappavoviruses display> 94% overall nt sequence similarity to the MIT-WI1 genome (Table 2). The MIT-WI1 genome sequence is closely related to the MSKCC strain (99.9%), the MKPV CI and MuCPV (98.2%), followed by the CPV XJC (94%). Compared to the MKPV CI genome, the genomes of MIT-WI1, MSKCC and MuCPV NYC strains contain a deletion of dinucleotides AA at positions 495 and 496 (Figure 2). This deletion is predicted to be silent due to its location downstream of the N-terminal coding region of alternatively spliced NS2 ORF, and has no apparent effect on its downstream gene transcription. The 4262 nt genome of TdCPV2 detected in faecal samples of Tasmanian devils displays 74–75% nt sequence similarity to MKPV/MuCPV strains. Phylogenetically, the nt sequence of the MIT-WI1 genome is most closely related to MKPV MSKCC (sFigure3A).

To further examine how the genomic divergence affects the aa compositions of predicted genes among these MKPV/MuCPV strains, the deduced aa sequences of each ORF were compared (Table 3). Generally, there are 98-100% similarity in the predicted p10, p15, NS1, NS2 and VP1 sequences among MKPV strains, which also share 60-82% aa sequence homology with the corresponding genes in TdCPV2. In line with the nucleotide sequence similarity, the aa sequences of the MIT-WI1 p10, NS1, NS2 display the highest similarity to their counterparts encoded by MKPV MSKCC. The MIT-WI1 p15 and VP1 are equally similar in their aa sequences to those in the genomes of MKPC CI, MSKCC and MuCPV NYC. In addition, the 387 aa sequence of the CPV XJ NS1 displays 98% similarity to the corresponding NS1 regions of MKPV MIT-WI1, CI, MSKCC and MuCPV. Of note, the MSKCC VP1 sequence is identical to that in the MKPV CI. Phylogenetic analyses indicate that the NS1 and VP1 aa sequences of MIT-WI1 are most closely related to MSKCC and MuCPV NYC, respectively (sFigure 3B and 3C).

**Table 3.** Amino acid sequences (aa) similarities of the corresponing proteins between MIT-WI1 and other known MKPV/MuCPV.

Strain	% p10 (98 aa)^a^	% p15 (130 aa)^a^	% NS1 (659 aa)^a^	% VP1 (496 aa)^a^	% NS2 (295 aa)^a^
MKPV MSKCC	100	100	99.7	99.8	100
MKPV CI	98.9	100	98.8	99.8	99.2
MuCPV NYC	N.A.	100	98.5	99.8	99.2
MuCPV XJC	N.A.	N.A.	98.4 (387aa, partial)^b^	N.A.	N.A.
TdCPV2 Aus	N.A.	78.6 (132 aa)^b^	72.1	60.7 (494 aa)^b^	81.2 (294 aa)^b^

Note: aa: amino acids; N.A.: not appliable.

^a^Parenthesis, numbers of aa deduced in the corresponding genes of the MKPV MIT-WI1 genome

^b^Parenthesis, numbers of deduced aa in the respective genes different from the MKPV MIT-WI1.

### Development of diagnostic PCR, qPCR assays for detecting MKPV

**Figure 3.**
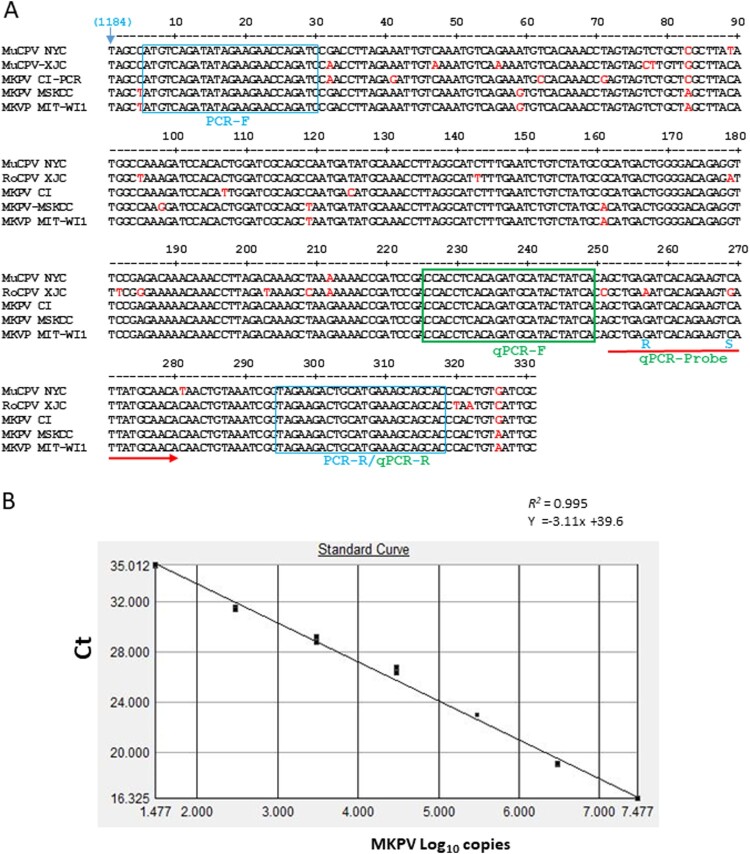
Development of PCR assays for detection of MKPV/MuPCV. A, *NS1* regions of 331 nt among known MKPV/MuCPV strains were aligned with the DNASTAR Lasergene 13 package. The nucleotide sequences of conserved PCR primers and qPCR primers/probe were identified. Letters in red represent degenerate nucleotides distinct from the conserved ones. Number in parenthesis corresponds to the genomic position of MKPV CI. B, Validation of the MKPV qPCR assay. Values of the linear regression *R^2^* and interception demonstrates optimal amplification efficiency for the assay; its detection limit is ≤ 10 copies.

Given that MKPV infection causes IBN in adult and aged immunocompromised mouse strains and appears to be prevalent in mice housed at animal facilities in academic institutions and wild rodents [10], universal PCR and qPCR assays are needed for detecting MKPV strains. Due to the presence of sequence divergence in previously described PCR primers that may affect the accuracy of MKPV detection, new primers MKPV-rF and –rR for regular PCR and a set of primers and probe for qPCR conserved among known MKPV strains were generated from the *rep* (NS1) sequence (Figure 3(A)). PCR amplification with newly designed MKPV-rF and –rR, of which at least one primer did not display significant nt sequence identity to mouse chromosomal DNA or other microbial genomes except for the MKPV/MuCPV genomes deposited in the GenBank database using the Blastn search and genome comparison among MKPV, MVM (NC_001510.1) and MPV1 (U12469.1) with the DNAStar Lasergene 13 package, produced a 314 bp product from faecal and kidney DNA of mice with IBN but not from healthy mice, confirming PCR specificity. For MKPV qPCR, a 10-fold standard curve of 10^7–10 was generated from the recombinant plasmid containing the target region (described in Materials and Method). Linear regression and interception of the qPCR assay were 0.99 and at ∼Ct 40 respectively, showing its dynamic detection range and optimal amplification efficiency (Figure 3(B)).

### MKPV is present in multiple mouse strains/stocks at the WI animal facilities

To assess status of MKPV infection in the animal facilities at WI, the DNA templates were prepared from faecal samples and kidneys of several mouse strains including B6.Il2rRag2W (embryo-rederived), c-*Kit^W–sh^* mice, sentinel Crl:CD1(ICR) mice housed with MKPV-infected mice, NSG, and aged UTX^flo*x*^ mice; these mice were housed in different animal holding rooms. MKPV PCR was performed on these samples using the newly designed primers (Table 4). The MKPV DNA was detected in selected c-*Kit^W–sh^*, sentinel Crl:CD1(ICR), NSG and UTX^flo*x*^, but was absent in all of the samples from rederived B6.Il2rRag2W mice. The nucleotide sequences of the selected PCR products were identical to the corresponding region of the MIT-WI1 genome and also display 98-99% similarity to other known MKPV strains. In addition, the MKPV genome sequence excluding 5′ and 3′ ITR in NSG mice was determined by genome walking and was identical to MIT-WI1. These results demonstrated that MKPV MIT-WI1 is present in the selected mouse strains/stocks housed at the MIT-WI animal facilities.

**Table 4.** MKPV status in selected mouse strains at MIT-WI animal facilities.

Location	Genotype	Age (months)	Application	Sample type	MKPV ^+^ %^a^
WI-A	UTX^flox^	18–28	Experimental	Frozen Kidney	66.6% (4/7)^c^
WI-B	Crl:CD1(ICR)	3.5	Sentinal	faecal	66%(2/3)^c^
WI-B	C57BL/6 *c-Kit^W–sh^*	4–10	Experimental	Frozen Kidney	33% (1/3)^c^
	* *			faecal, pooled	5.5% (1/18^b^)
WI-C	* *				21.4% (3/14^b^)
WI-C	NSG	2.5–6		Frozen Kidney	71.4% (10/14)^c^
WI-D	B6.Il2rRag2W	3.5–22		FFPE Kidney	95.5% (23/24)
WI-E	*Rederived* B6.Il2rRag2W	8–10		Frozen Kidney	0 (0/14)

Notes: ^a^numbers in parenthesis represent number of MKPV^+^ per total sample numbers.

^b^Per cage.

^c^Sanger sequencing of select PCR products.

### Levels of MKPV are positively correlated with severity of IBN in immunocompromised mice

**Figure 4.**
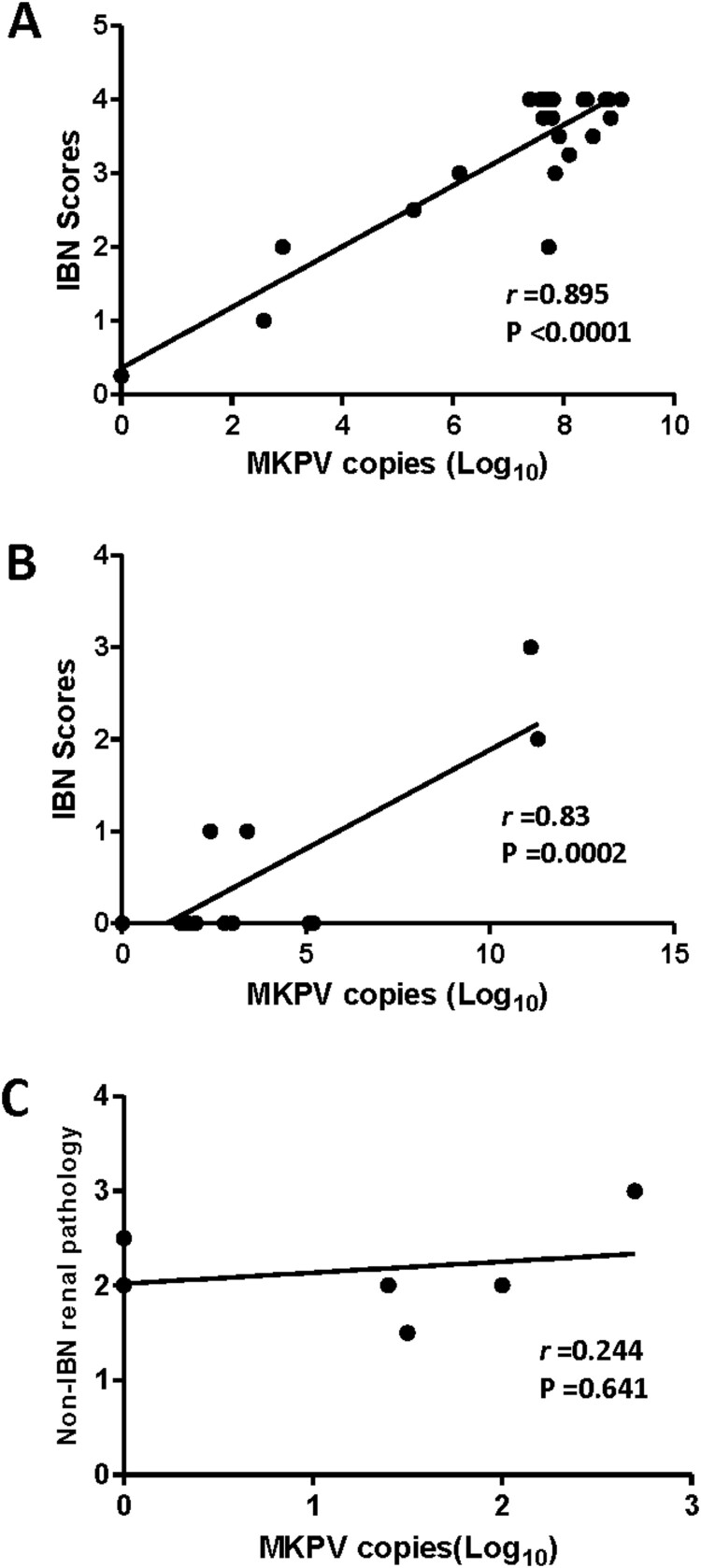
Abundance of MKPV in kidney of immunodeficient mouse strains was significantly correlated with severity of IBN. (A) B6.Il2rRag2W, (B) NSG and (C) UTX^flox^. Abundance of renal MKPV in mice was significantly correlated with severity of IBN in immunocompromised mice (A) and (B), but not with non-IBN renal pathology in immunocompetent mice (C). Pearson correlation was performed with a two-tailed test.

It has been documented that levels of MKPV in the mouse kidneys progressively increased over the ages of 60 to > 200 days with the highest levels being detected in mice with IBN [5]. We sought to determine the abundance of MKPV in the FFPE kidneys of B6.Il2rRag2W mice, and the frozen kidneys of NSG and UTX^flo*x*^ mice using the qPCR assay developed in this study (Figure 4). Of the 10 NSG mice infected with MKPV, 4 had lesions consistent with IBN and five had lesions in other organs secondary to sepsis*.* In addition, MKPV mRNA expression was also detected in the kidneys of IBN-affected, but not MKPV-negative NSG mice (sFigure 1C and D). Of the 6 UTX^flo*x*^ mice, 4 had low levels of MKPV (<1000 copies), but no IBN was noted in these mice (Figure 4(C)). However, all UTX^flo*x*^ mice had concurrent chronic degenerative changes in the kidneys (e.g. mesangioproliferative glomerulopathy) and tubular epithelial cell karyomegaly. There was a significant correlation between the abundance of MKPV and severity of IBN in B6.Il2rRag2W (Figure 4(A), *r *= 0.895, *P* < 0.0001). This result indicates that the increased abundance of MKPV contributed to more severe IBN in the kidneys of immunocompromised B6.Il2rRag2W. A similar correlation between severity of IBN and abundance of MKPV was observed in NGS mice (Figure 4(B), *r *= 0.82, *P* = 0.0002). This correlation needs to be further validated with additional IBN-affected NGS mice.

## Discussion

In this study, we identified and characterized a new strain of MKPV, MIT-WI1, in B6.Il2rRag2W mice with severe IBN that is similar to the MKPV-induced nephropathy recently noted in other immunocompromised mouse strains [4,5,10]. Infection with MKPV, a member of the provisional virus genus *Chapparvovirus*, can ultimately lead to renal failure in immunocompromised mice. Infection of mice with MKPV may represent a useful model for studying pathogenesis of virus-associated nephropathies and interstitial fibrosis in humans. In this study, we documented that IBN noted in MKPV-infected B6.Il2rRag2W mice share histopathological features consistent with polyomavirus-associated nephropathy, which is increasingly recognized as the most common viral complication in immune-suppressed human kidney transplant recipients [12,13].

The MIT-WI1 genome of 4440 nt in length contains a dinucleotide deletion compared with MKPV CI in the 5′-end region and shares an identical gene organization compared with that of the MKPV strains CI and MSKCC, and MuCPV NYC [5]. In addition, over 94% nt sequence similarity is shared among the complete or partial genomes of MKPV strains (MIT-WI1, MKSCC, CI), and MuCPV NYC and XJC identified in wild mice; these chapparvoviruses were isolated in diverse geographic locations and are distantly related to other isolates of *Chapparvovirus* [10,11]. Furthermore, MKPV DNA was detected in laboratory mice with IBN housed at the University of North Carolina (Chapel Hill, USA) and in Israel [10]. These lines of evidence indicate that this group of genetically related and geographically diverse chapparvoviruses constitutes a new virus species of *Chapparvovirus* according to the International Committee on Taxonomy of Viruses guidelines. Recently, Lee *et al*. proposed that selected chapparvoviruses including MKPV, TdCPV and CKPV (capuchin kidney parvovirus), which colonize kidneys and share similar gene organization with MKPV, could be classified into a new virus genus, *Nephroparvovirus* [10].

Phylogenetically, the MIT-WI1 genome is most closely related to MKPV MSKCC followed by MKPV CI and then MuCPV NYC based on the similarity of the overall nt sequences [5,6]. The phylogenetic relatedness between MKPV MSKCC and MIT-WI is also supported by the highest similarity in aa sequences of p10, p15 NS1 and NS2 shared by these two strains compared with other MKPV/MuCPV strains. Significant similarity in the nt sequences (>94%) and deduced ORF aa sequences (>98%) of the MKPV/MuCPV genomes among strains identified from different geographic regions including Australia, China and USA suggests that the genome structures and functions among the MKPV strains have been highly conserved. It is worth noting that there is a dinucleotide (AA) insertion in the 5′-terminal region of the MKPV CI genome from Australia compared with MKPV MIT-WI1, MSKCC and NYC from northeastern USA. Only MKPV CI has been identified in mice which were all descendants of the *Rag1^tm1Bal^* strain imported from northeastern USA in the mid-1990s into the Centenary Institute facilities. It was, therefore, suggested that MKPV CI was likely introduced into Australia by the imported *Rag1^tm1Bal^* mice [10,14]. If this speculation is true, this AA insertion as well as additional genetic divergence in the MKPV genomes could have occurred and evolved into the current CI genome in Australia. Indeed, a sub-strain of CI containing an extra C in its 3′ ITR was identified in one mouse, suggesting that divergence of the MKPV genomes has slowly evolved [10].

We found that both higher abundance of the MKPV genome and MKPV mRNA expression were detected in the IBN-affected kidneys of B6.Il2rRag2W and NSG mice compared with those without IBN. This result is consistent with previous findings that MKPV infection is linked to chronic tubulointerstitial nephropathy and kidney fibrosis in aged mice, predominantly in immunodeficient mouse strains. It is also important to note that MKPV can be detected in urine, faeces, blood and other organs, such as liver, and spleen, and occasionally in caecum, and urinary bladder [5,10]. Interestingly, a recent study reported that MKPV RNA was only detected in murine kidney but not in murine liver or spleen, suggesting that the kidney is more permissive for MKPV transcription enabling MKPV to replicate and induce pathologic lesions in kidneys [10]. A higher incidence and more severe IBN in concert with increased abundance of MKPV in aged immunodeficient mice suggest that MKPV-induced IBN partially results from progressive dysregulation of certain immune factors controlling propagation of MKPV in affected kidneys during aging. This notion is supported by our finding that MKPV was significantly lower in abundance and did not induce IBN in aged immunocompetent UTX^flo*x*^ mice. Future studies are needed to elucidate how MKPV is recognized, internalized and spread in the kidneys of infected mice.

By use of newly developed PCR, MKPV DNA was detected in faecal and kidney samples from several mouse strains/stocks including immunocompromised mice (NSG and B6.Il2rRag2W) with IBN and immunocompetent mice (Crl:CD1(ICR)*,* UTX^flo*x*^ and *c-Kit^W–sh^*) without IBN. Our finding that sentinel Crl:CD1(ICR) mice cohoused with MPKV^+^ mouse strains were also MKPV-positive demonstrates that MKPV can be transmitted to outbred sentinel mice via dirty bedding. MKPV^+^ percentages among the samples were highly variable, ranging from 10 to 70% and appeared to be dependent on the mouse strain background immune status and ages of mice. By surveying 3517 faecal samples collected from mice housed at 78 biomedical research institutions over a seven-month period, Lee *et al.* reported that an overall prevalence of MKPV was 5.1% [10].

In the present study, the 314 nucleotide sequences of the PCR products from Crl:CD1(ICR) and UTX^flox^, and the nearly complete MKPV genome sequence present in NSG are identical to the MIT-WI1 genome identified in B6.Il2rRag2W mice and also display >98% similarity to other MKPV strains previously described [5,6]. These results suggest that the laboratory mice at the WI facilities were likely infected with a single MKPV strain. Interestingly, kidneys collected from all of 14 rederived B6.Il2rRag2W mice at ages of 8–10 months were negative for MKPV via qPCR, suggesting that embryo rederivation could be utilized to eliminate MKPV. However, a prospective study on the status of MKPV infection in embryos of IBN-affected mice is required for establishing whether MKPV can be eliminated via embryo rederivation.

Four mouse parvoviruses, including MPV-1–3 and MVM, are closely related to MKPV in their genome structures (the 5′- and 3′-end hairpin structures, ORFs for NS1and VP1) [10,15]. Natural MKPV infection can cause severe morbidity and mortality, while natural infections of the other murine parvoviruses are generally asymptomatic in both immunocompetent and immunocompromised mice [1]. It is worth noting that the newly identified accessory proteins P10 and P15 of MKPV are apparently absent in the genomes of the other murine parvoviruses. The role of these proteins in MKPV pathogenicity needs to be further characterized.

In summary, the complete genome sequence of MKPV MIT-WI1 determined in this current study is most closely related to MSKCC phylogenetically. The gene organization and 5′/3′-ITR structures of the genomes of known MKPV/MuCPV strains are highly conserved. PCR and qPCR assays conserved among known MKPV/MuCPV have been developed and can be utilized for monitoring the status of MKPV infection in mice. Based on our study, MKPV is present in a subset of mouse colonies housed at the WI facilities, and increased abundance of MKPV is strongly correlated with severity of IBN in aged immunodeficient mouse strains. MKPV infection in experimental mouse strains can be a confounding factor and a serious argument can be made to exclude MKPV from mouse colonies used in biomedical research.

## Supplementary Material

MKPV-MS-sFigure_3-R2.tif

sFig-2.tif

sFigure_1.tif
