# Supplementary figures and images for: Identification of a new strain of mouse kidney parvovirus associated with inclusion body nephropathy in immunocompromised laboratory mice
Source: Emerg Microbes Infect. 2020 Aug 10;9(1):1814–23. doi: 10.1080/22221751.2020.1798288 (PMC7473309; doi:10.1080/22221751.2020.1798288)

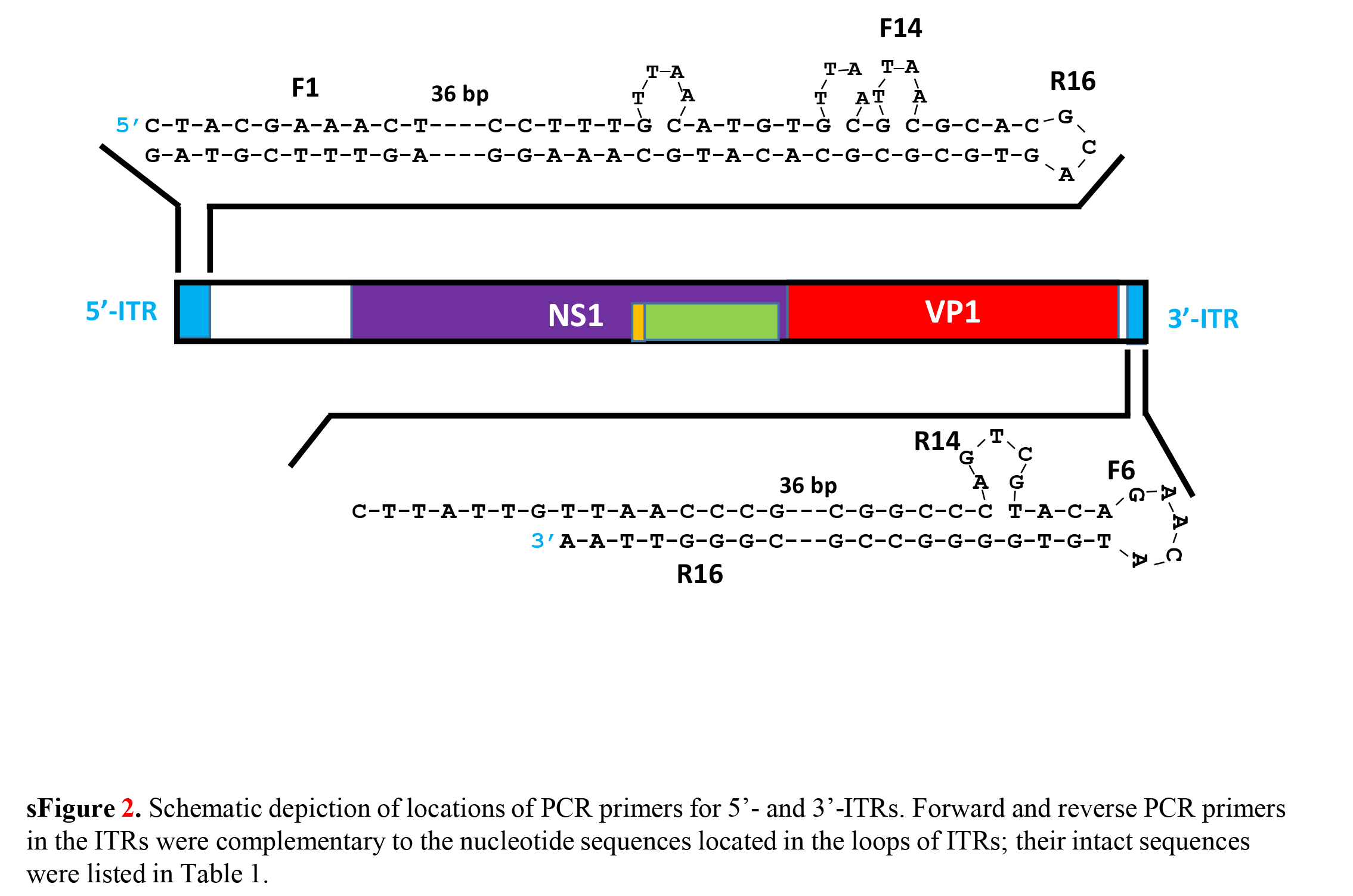

Supplement: sFig-2.tif [file TEMI_A_1798288_SM7979.tif]

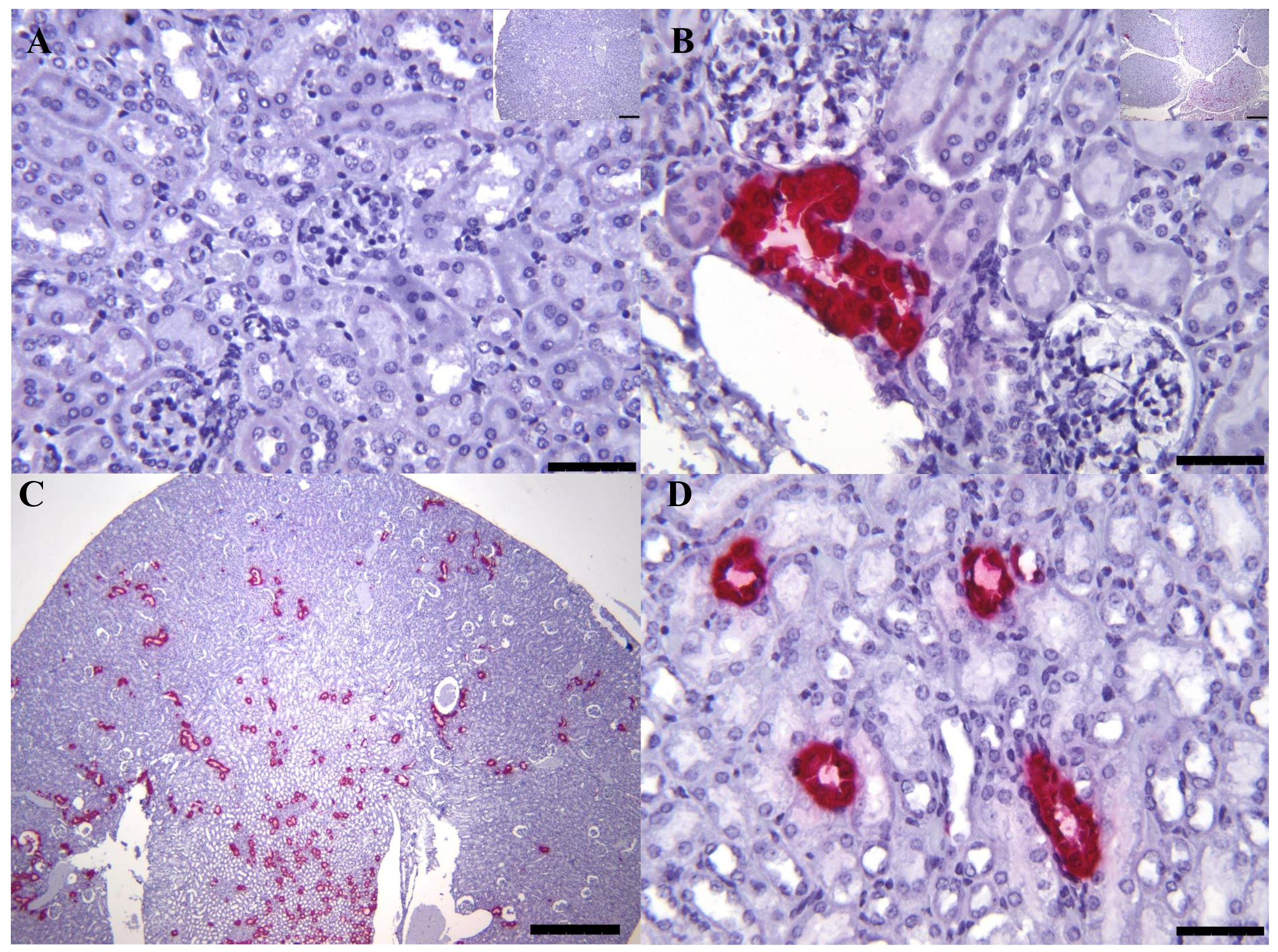

Supplement: sFigure_1.tif [file TEMI_A_1798288_SM7978.tif]
